# Sequence-specific delayed gains in motor fluency evolve after movement observation training in the absence of early sleep

**DOI:** 10.1038/s41598-024-53004-4

**Published:** 2024-02-18

**Authors:** Rinatia Maaravi-Hesseg, Sigal Cohen, Avi Karni

**Affiliations:** 1 Sagol Department of Neurobiology, University of Haifa, 3498838 Haifa, IL Israel; 2 E. J. Safra Brain Research Centre for the Study of Learning Disabilities, University of Haifa, 3498838 Haifa, IL Israel

**Keywords:** Consolidation, Cognitive neuroscience

## Abstract

Following physical practice, delayed, consolidation-phase, gains in the performance of the trained finger-to-thumb opposition sequence (FOS) can be expressed, in young adults, only after a sleep interval is afforded. These delayed gains are order-of-movements specific. However, in several perceptual learning tasks, time post-learning, rather than an interval of sleep, may suffice for the expression of delayed performance gains. Here we tested whether the affordance of a sleep interval is necessary for the expression of delayed performance gains after FOS training by repeated observation. Participants were trained by observing videos displaying a left hand repeatedly performing a 5-element FOS. To assess post-session observation-related learning and delayed gains participants were tested in performing the observed (trained) and an unobserved (new, the 5-elements mirror-reversed) FOS sequences. Repeated observation of a FOS conferred no advantage to its performance, compared to the unobserved FOS, immediately after practice. However, a clear advantage for the observed FOS emerged by 12 h post-training, irrespective of whether this interval included sleep or not; the largest gains appeared by 24 h post-training. These results indicate that time-dependent, offline consolidation processes take place after observation training even in the absence of sleep; akin to perceptual learning rather than physical FOS practice.

## Introduction

Skilled motor performance, "how to", “what to do” knowledge, is usually achieved through actual physical practice^1–3^. However, the repeated observation of a task being performed can also lead to subsequent specific performance gains; observation facilitating learning processes (e.g.,^4–7^). There is a debate whether motor skills learned by repeated observation are represented by the same neural substrates and even depend on the same processes of plasticity as those underlying motor skill acquired by actual physical practice. For example, the primary motor cortex (M1), among other brain areas, was found to be activated more intensely^8–10^ or even exclusively^11,12^ during actual practice. However, even given M1 engagement in learning novel motor tasks by observation^5,6,13–15^, this does not necessarily indicate that the same units in M1 are engaged for mnemonic representation by observation or actual training (e.g.,^16,17^). The results of a study of interference to motor memory consolidation suggest that skill gained from observation and skill attained through physical practice may not strictly overlap^18^. It was shown that the expression of delayed gains in the performance of a physically trained finger-to-thumb opposition-movement sequence (FOS) could be blocked by subsequent training on a different FOS, only if the second sequence was physically trained, but not when training on the second sequence was by observation; when the second FOS was trained by observation the participants showed robust overnight gains in performance for both the initially and the second trained sequences^18^.

Here we addressed the possibility that the mnemonic processes engaged in learning through observation may differ from the processes engaged in learning from actual practice, by considering that the former may be more akin to mnemonic processes of the type engaged in some forms of perceptual learning. In young adults, motor skill learning consolidation processes, specifically the processes sub-serving the emergence of delayed gains in the performance of a specified FOS, require sleep in order to evolve in the hours following practice^19–22^. However, the expression of delayed gains following perceptual learning is not dependent on the affordance of a post-practice interval with sleep; the passage of time suffices for completing these processes^23,24^. Thus, we tested whether the consolidation processes of learning a movement sequence (FOS) by repeated observation are sleep dependent, as is the case of actual physical practice on a FOS, or, as in the case of several perceptual learning paradigms, can evolve with the passage of time, independently of the availability of sleep in the post-learning interval.

A seminal study suggested that sleep closely following training may be necessary in order to express sequence-specific performance gains after the learning of a finger (key press) movement sequence by observation; only evening trained groups (sleep following) had gains 12 h later, sleep that came after more than 12 h of wake resulted in no benefits^25^. However, because the performance of observed and unobserved sequences was tested in different groups of participants, and baseline performance testing by necessity affords actual physical practice, the effect of group differences (at baseline) could not be ruled out. Further support for a possible beneficial effect of early post-training sleep in training by observation, comes from a recent study of participants undergoing multiple sessions (3 weeks) of video-clip based action observation training (additional mental imagery practice was included)^26^. It was found that manual action observation training (the video-clips observed in training presented transitive daily tasks performed with the right upper limb) had improved the participants’ manual dexterity (tested in tasks other than the tasks used in training) and that the gains in manual dexterity were larger for participants trained at evening (followed by early sleep) compared to those undergoing the same training at least 12 h before sleeping^26^. However, each time-of-day condition was tested in a different group of participants, and, as the authors acknowledge, the potential influence of circadian rhythms on observation or motor imagery could not be ruled out. A recent study of typing-sequence learning^27^ found no significant delayed gains in performance after training by observation, even when a brief interval of daytime (nap) was afforded, a result that may indicate that the length of the post-training interval rather than sleep may be important.

**Figure 1:**
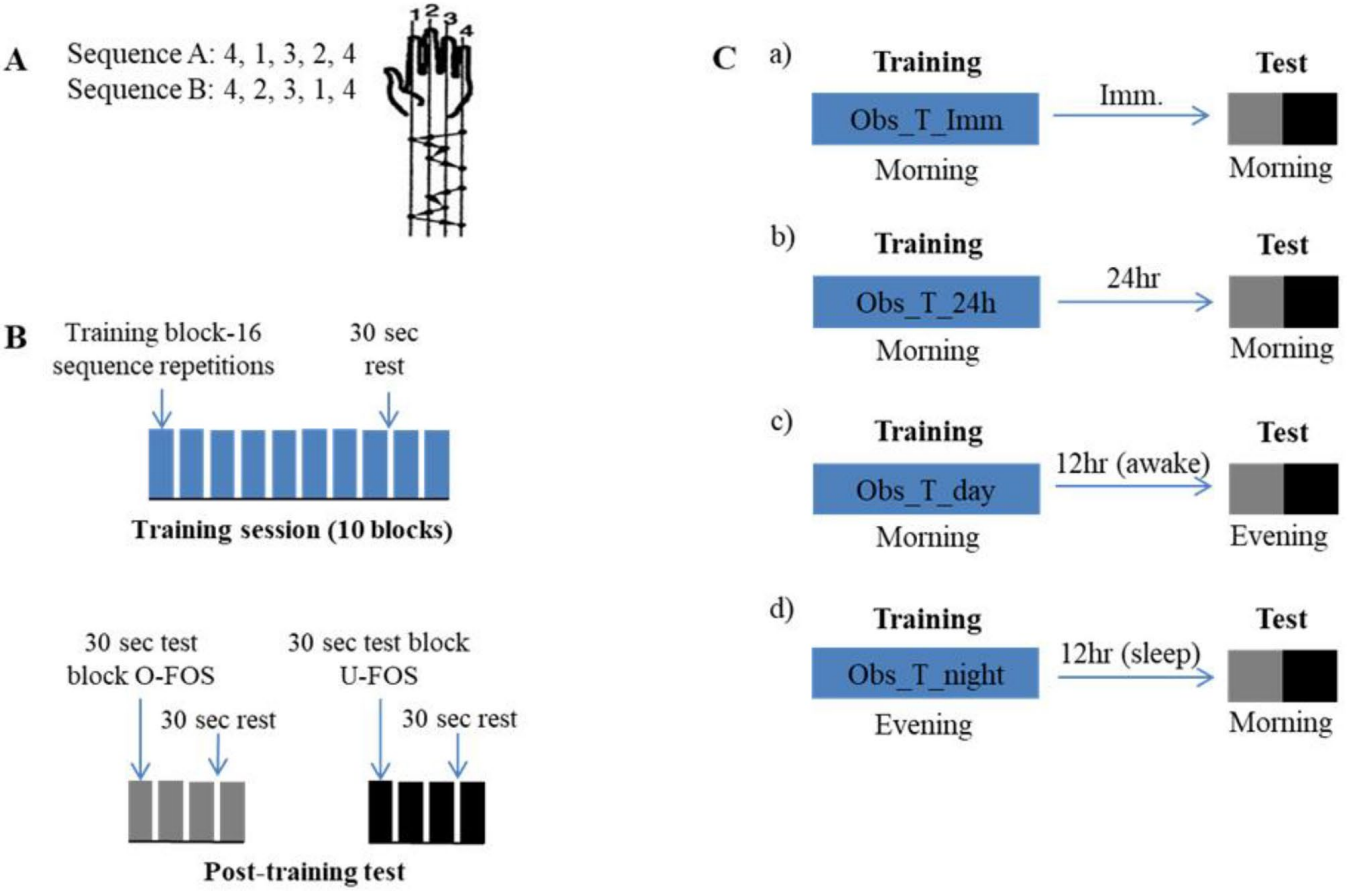
The finger-to-thumb opposition sequence (FOS) task (**A**), the training protocol (**B**) and the overall study design (groups-conditions) (**C**). (**A**) Two five-element sequences, each the mirror-reverse of the other, were used. (**B**) The training protocol consisted of viewing 160 repetitions of the assigned FOS (10 video-clips with 16 FOS repetitions in each) tapped by a person’s left hand. (**C**) Three groups observed the to-be-learned FOS (O-FOS) in a morning session: (a) group Obs_T_Imm was tested immediately after the training session; (b) group Obs_T_24h was tested 24 h after the session, overnight; (c) group Obs_T_day was tested 12 h after the session. Group Obs_T_night observed the to-be-learned FOS in the evening and was tested 12 h later, overnight (d). In all 4 groups, participants were first tested on the observed sequence (O-FOS) and immediately after on the untrained (mirror-reversed) movement sequence (U-FOS).

In the current study a within-subject design was used to directly address the question of whether the 12 h post-training interval must include a sleep period, after FOS practice solely by repeated observation, in order to enable the expression of delayed performance gains for the observed (trained) FOS (i.e., the expression of delayed gains specific to the performance of the repeatedly observed movement sequence). The FOS protocol affords two comparable sequences (5 finger-to-thumb opposition movements), sharing the same movements and complexity but differing in syntax (the order of the component opposition movements is mirror reversed in the two sequences) (Fig. 1A). Physical and observation training on one FOS results in immediate gains for both sequences, but specific practice on any one of the sequences results in the expression of delayed gains solely for the trained FOS^18,28^. Participants practiced one of the sequences by observing short video clips of a left hand repeatedly performing the task from the viewpoint of an observer looking at his/her own hand; the other FOS served as the unpracticed task condition, a control (Fig. 1A,B).

Three groups of participants trained on one of the FOSs by observation (O-FOS) in the morning and differed only in the time interval that elapsed between the training session and the performance tests: participants in the Obs_T_imm group were tested immediately after training; participants in the Obs_T_24h group were tested 24 h later; participants in the Obs_T_day group were tested 12 h after the observation session, in the evening of the same day (Fig. 1Ca–c). However, the participants of a fourth group (Obs_T_night group) trained by observation in the evening and were tested 12 h later, overnight, on the next morning (Fig. 1Cd). Immediately after being tested on the observed sequence, all participants were also tested on the mirror-reversed (untrained) movement sequence (U-FOS) (Fig. 1B).

## Results

**Figure 2:**
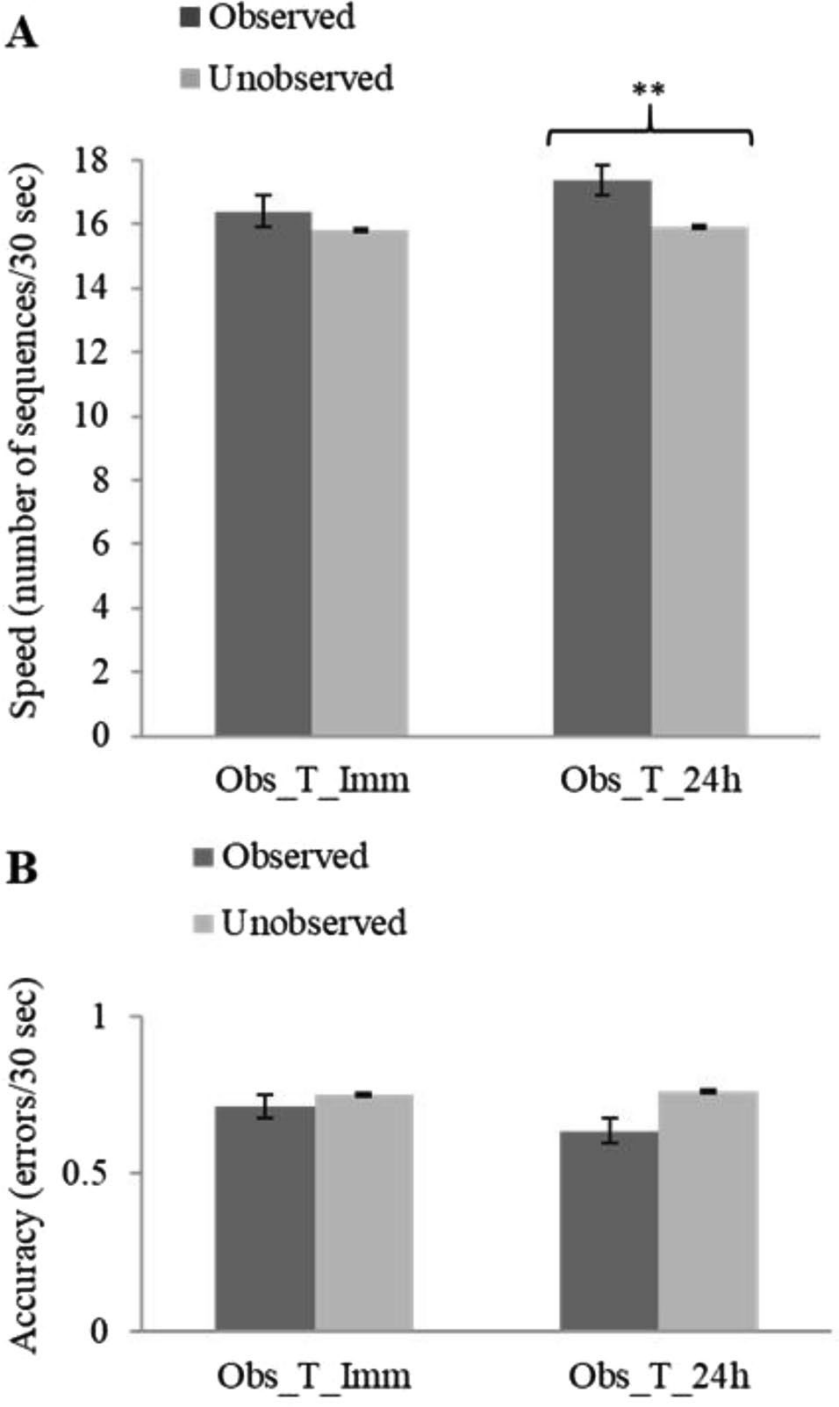
Participants’ mean speed (**A**) and accuracy (**B**) in executing the O-FOS and U-FOS in the immediate (Obs_T_Imm) and overnight (Obs_T_24h) tests. Bars represent standard error of the mean.

First, we established that the training-by-observation protocol could induce consolidation processes expressed as delayed gains for the observed, practiced, O-FOS vis-à-vis the unobserved U-FOS. The analyses were based on within-subject comparisons; comparing the performance of the individuals’ O-FOS to U-FOS performance, in each group (test, time-point). Paired-samples t-tests, one for performance rate (speed; the number of correct sequences tapped in the test interval) and one for accuracy (number of incorrect sequences tapped in the test interval) were used for comparing the performance of participants on the two sequences (O-FOS, U-FOS) in the group tested immediately post-training (group Obs_T_imm) and in the group 1st tested at 24 h post-training (Obs_T_24h) (Fig. 2). As can be seen in Fig. 2 there were no significant differences in the performance of the two sequences (O-FOS, U-FOS) in the group tested immediately post-training (Obs_T_imm), in either speed [t(19) = 1.42, *p* = 0.17, d = 0.33] or accuracy [t(19) = 0.03, *p* = 0.978, d = 0.006]. However, participants that were tested at 24 h post-training (Obs_T_24h, afforded a night’s sleep) showed a significant advantage in performing the observed FOS, vis-à-vis the U-FOS, in terms of speed of performance [t(19) = 3.78, *p* < 0.01, d = 0.844] with no cost in accuracy (no difference in number of errors compared to the U-FOS; in fact, on average, somewhat fewer errors in the O-FOS) [t(19) = − 0.97, *p* = 0.344, d = 0.23], (Fig. 2A,B). Note that participants were very accurate and errors were rarely committed in any of the study conditions. Thus, by 24 h after training by observation there were clear delayed gains expressed for the O-FOS.

**Figure 3:**
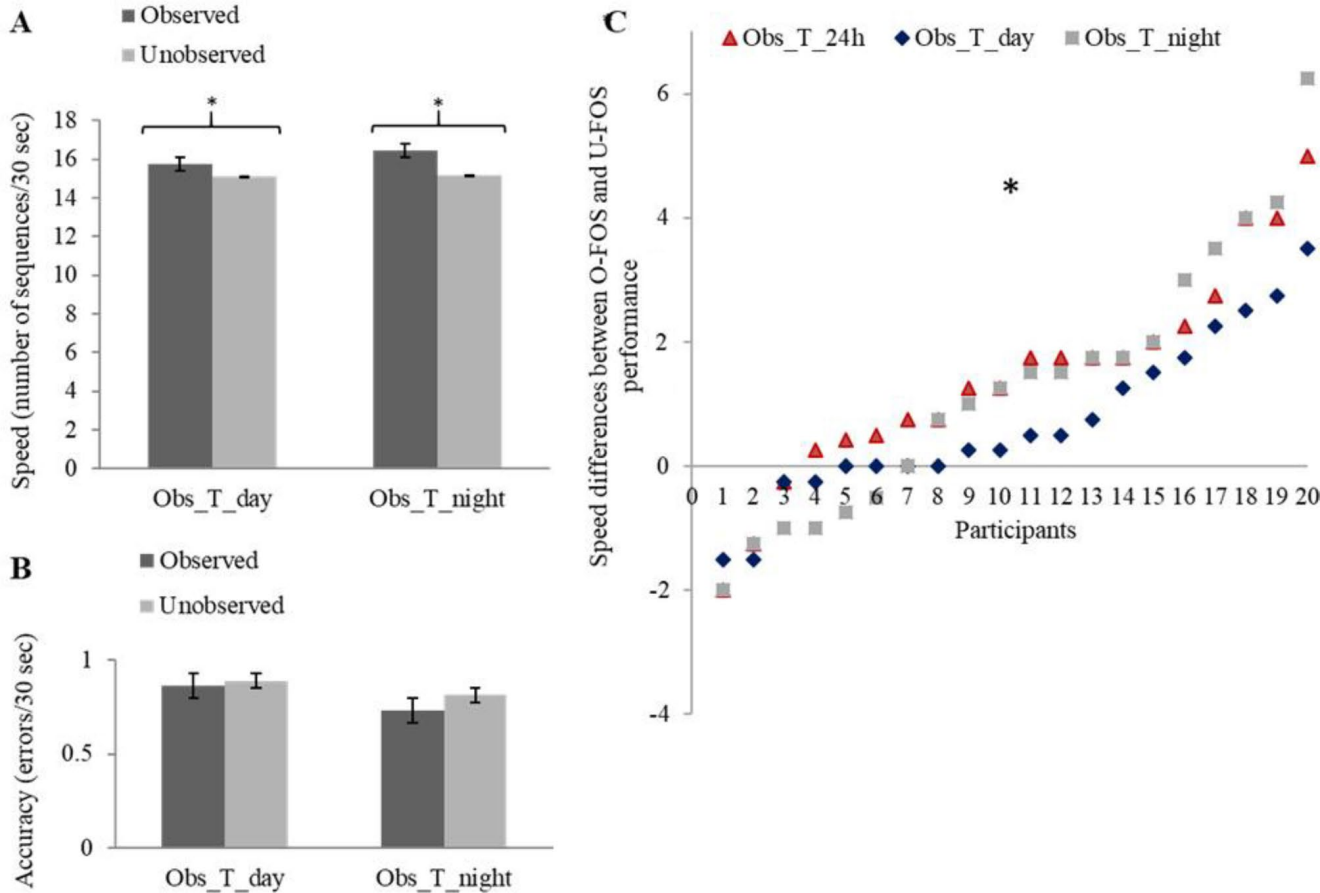
Group and individual performance on the O-FOS compared to the U-FOS after the two 12-h intervals (groups Obs_T_day; Obs_T_night). (**A**, **B**) Speed and accuracy after a delay interval of 12 h, with and without sleep. Bars represent the standard error of the mean. (**C**) Individual data, the difference (delta) in performance speed for the O-FOS versus U-FOS; the results of the two 12-h interval groups (Obs_T_day; Obs_T_night) are shown in comparison to the results of the 24-h interval group (Obs_T_24h).

Smaller but significant delayed gains in the performance of the O-FOS vis-à-vis the U-FOS were expressed also by the participants that were tested after a shorter, 12 h, interval of daytime activities (Obs_T_day); i.e., when sleep was not afforded in the interval. Paired samples t-tests comparing the observed and unobserved sequences in the Obs_T_day group showed significant advantage to the O-FOS over the U-FOS in terms of performance speed [t(19) = 2.63, *p* < 0.05, d = 0.594] with no cost in accuracy [t(19) = − 0.29, *p* = 0.772, d = 0.066] (Fig. 3A,B). This pattern of results underscored the notion that early post-learning sleep may not be a necessary factor for the progression of consolidation processes after training by observation. Nevertheless, only 12/20 participants of the Obs_T_day group showed an O-FOS advantage compared to 17/20 in the Obs_T_24h (Fig. 3C), and the O-FOS specific delayed gains attained in the Obs_T_day group were on the order of 51% of the group-average gains expressed by participants of the Obs_T_24h.

Group Obs_T_night was afforded training in the evening and subsequently a 12 h interval before testing. This group was run to test whether the larger and more consistent (across participants) gains expressed in the Obs_T_24h group were the result of the extended time interval from training to testing (allowing more time for consolidation processes) or reflected the beneficial effect of the affordance of a night’s sleep in the post-training interval. To this end, paired sample t-tests (for speed of performance and for accuracy, separately) comparing the performance of the O-FOS to the U-FOS were run for the Obs_T_night group. The results showed a significant advantage for the O-FOS, vis-à-vis the U-FOS, in terms of speed [t(19) = 2.75, *p* < 0.05, d = 0.614] (Fig. 3A) and no difference in accuracy [t(19) = − 0.68, *p* = 0.507, d = 0.151] (Fig. 3B). 13/20 participants of the Obs_T_night group showed an O-FOS advantage and at group level, this observation-related advantage was on average 86% of the relative gains expressed in the Obs_T_24h group. However, a t-test comparing the O-FOS to U-FOS difference (delta delayed gains) in the two groups, showed no significant group differences (t(38) = 0.339, *p* = 0.737, d = 0.107; t(38) = − 0.234, *p* = 0.816, d = 0.075, speed and accuracy, respectively). Also, the gains for the O-FOS in the Obs_T_night were not significantly different from those obtained by participants in the Obs_T_day group (t-test comparing the O-FOS to U-FOS difference (delta delayed gains), t(38) = − 0.941, *p* = 0.353, d = 0.298; t(38) = 0.39, *p* = 0.699, d = 1.234, speed and accuracy, respectively).

We also ran a repeated measures ANOVA (GLM analysis) comparing the performance speed of the Obs_T_night and Obs_T_day groups, as a between subject factor, for the two sequences (O-FOS, U-FOS) as a within subject factor. The results showed no significant group effect [F(1,38) = 0.105, *p* = 0.747, η^2^ = 0.064] nor a significant interaction between the groups and sequences [F(1,38) = 1.137, *p* = 0.293, η^2^ = 0.18]. There was a significant advantage for O-FOS performance compared to U-FOS performance [F(1,38) = 12.956, *p* < 0.01, η^2^ = 0.939]. Thus, the results indicated that while sleep was not a necessary condition for expressing delayed gains in the post-training interval, the length of time afforded for consolidation processes was a factor in determining the aftermath of FOS learning from observation.

A repeated measures two-way ANOVA (3X2) comparing the performance speed of the three delayed-test groups in the two sequences (O-FOS, U-FOS, as a within subject factor) showed no significant Group effect [F(2,57) = 0.614, *p* = 0.545, η^2^ = 0.148] as well as no significant interaction between the groups and sequences [F(2,57) = 0.977, *p* = 0.383, η^2^ = 0.212]. However, the effect of sequence, i.e., the observation training, was found to be significant, with an overall advantage for the observed sequence [F(1,57) = 25.995, *p* < 0.001, η^2^ = 0.999]. The gains in speed were not at the cost of accuracy; a similar analysis of the accuracy of performance showed no difference between the groups in the number of errors committed for the O-FOS vis-à-vis the U-FOS [F(1,57) = 1.397, *p* = 0.242, η^2^ = 0.213].

A nonparametric test (chi-square) was performed to compare the number of participants in each group that showed an advantage for the O-FOS as compared to the U-FOS in each group, i.e., after the post-learning interval afforded in each condition. The chi-square test was used to test whether the ratio of participants who showed an O-FOS advantage (over the U-FOS) after the observation session (gainers) vis-a-vis the participants that did not show an O-FOS advantage (non-gainers), differed from a 50/50 ratio, in any of the 4 groups tested. The proportion of participants showing an advantage for the O-FOS compared to the U-FOS (gainers) was significantly above 50:50 only in the Obs_T_24h group (17/3) [χ^2^ (1,20) = 9.8, *p* < 0.01]; there were significantly more gainers than non-gainers when testing was delayed for 24 h (Fig. 3C). The proportion of gainers to non-gainers was not significantly different from 50:50 in the Obs_T_night (13/7) [χ^2^ (1, 20) = 1.8, *p* = 0.18] and the Obs_T_day (12/8) [χ^2^ (1, 20) = 0.8, *p* = 0.371] groups (Fig. 3C); again indicating that the shorter (12 h) post-training intervals, irrespective of whether sleep was included, were comparable in advancing the O-FOS performance.

## Discussion

Taken together, the current results underscore the notion that the practice of a sequence of finger-to-thumb opposition movements by repeated observation can result in a significant advantage in the subsequent performance of the observed movement sequence; observed-sequence specific performance gains evolved in the hours that followed the observation session. These gains were specific to the observed sequence; thus the advantage in the performance of the previously observed sequence over the performance of a novel, previously unobserved movement sequence of equal length and structure. Delayed, consolidation phase, gains for the O-FOS were expressed in some participants already at 12 h post-training, but a 24 h post-learning interval was found superior in enabling most participants to express delayed performance gains. However, the current results also indicate that sleep afforded in the post-session interval, may not be necessary for the subsequent expression of consolidation phase gains (sequence specific “how to” knowledge) following practice by observation. Rather the length of the interval afforded after the practice session may be more critical for the advancement of consolidation phase gains.

The passage of time after practice has been shown to be important to the consolidation of “how to” knowledge attained via actual, physical, practice in the same task addressed in the current study; there is also data indicating that larger delayed gains may be expressed by 48 h compared to 24 h post-training on a given FOS (e.g.,^28^). However, in young adults, the affordance of sleep in the post-learning interval, either immediately or even 12 h and later after training, was shown to be a critical factor in the advancement and expression of consolidation phase gains after actual, physical, practice^20,28^. A recent study of key press (typing movements) sequence learning^27^ reports that a 3 h long interval containing ~ 90-min of daytime nap did not result in significant delayed gains in the performance of the trainees, irrespective of whether participants had physical or mental practice, or trained by observation. This pattern of results suggests that a brief post-training interval, even one that includes an interval of daytime sleep, may not suffice for participants to express delayed gains in the performance of an observed sequence of finger movements. This is in line with our hypothesis that the length of the post-training time interval, rather than the affordance of sleep per se, may be critical for mnemonic processing after observation learning. Note, however, that in children, before puberty, the expression of delayed gains after physical motor practice of a FOS was found to advance without a need for sleep^29^.

Robust delayed improvements in task performance, evolving in the post-learning interval even before sleep was afforded, have been described in young adults afforded training in several perceptual learning tasks (e.g.,^23,24,30,31^). Thus, for example, young adults training in a visual texture target discrimination task^24^ or in an auditory syllable-in-noise discrimination task^23^ showed the emergence of significant delayed gains in the performance of the trained tasks a few hours post-training, although no sleep was afforded in the interval. In both studies, in fact, increasing the interval between the practice session and the subsequent test for performance (and a night sleep was included) led to more participants expressing larger delayed gains; in the Ari-Even Roth et al.^23^ study the delayed gains in the performance of auditory syllable-in-noise discrimination tended to almost double after 24 h compared to 6–8 h post-training. Similarly, Kristoffer et al.^32^ used a visual acuity task to test whether perceptual learning is possible without post-training sleep and found that the contribution of sleep to the improvement of performance between sessions was minimal. Thus, the current results suggest that the acquisition, via observation, of specific “how to” knowledge pertaining to a movement sequence may proceed in a time-course more similar to that of perceptual skill consolidation, rather than under the additional constraints that apply, after puberty, to skill memory consolidation after (actual) motor practice^33^.

Although not directly addressed in the current study, it may be of relevance to note a possible additional difference pertaining to the involvement of sleep in the consolidation of ‘how to’ knowledge acquired perceptually, compared to ‘how to’ knowledge acquired through physical motor practice. The sleep stages that are deemed critical for advancing consolidation processes in motor skill acquisition may differ from the ones implicated in consolidating perceptual learning. Delayed gains in the performance of physically trained motor sequences are correlated with non-REM sleep (SWS or spindles at Stage 2 sleep)^34–38^. However, delayed gains in perceptual tasks (although these can evolve also independently of sleep) may require periods of REM sleep (e.g.,^24,39–41^) perhaps due to the similarities between REM sleep and the wake state, such as rapid, low-voltage desynchronized cortical activity^42,43^ and the engagement of the cholinergic system^44–48^.

There were some indications in the current findings that a 12 h long post-training interval with sleep included, may benefit trainees more than a comparable interval with no sleep afforded (specifically, on average larger delayed gains in the Obs_T_night group compared to the Obs_T_day group). It cannot be ruled out that the lack of statistically significant differences between the Obs_T_night and Obs_T_day groups may reflect insufficient power (small groups, large individual differences). The Obs_T_night advantage, however, may be the result of the difference in the time-of-day in which the two groups trained. The results of a recent study, addressing the effects of training by mental imagery, indicated a possible time-of-day effect^49^; participants had superior delayed gains when training was afforded in the afternoon compared to after a morning session. An advantage for evening practice was described in FOS practice by young adults with ADHD^50^. Future studies can address this issue as pertaining to observation learning, especially given the possibility that practice late in the day may decrease the potential for interference from everyday activities in the consolidation of newly learned movement sequences (e.g.,^51^).

In the current study, a difference in the performance of the two finger opposition sequences did not emerge immediately after observation. However, by 24 h post-training participants’ performance of the observed FOS was superior to that of the untrained FOS, replicating previous results^18^. Thus, the current results point to a “how to” memory consolidation phase triggered by the repeated observation of movement sequences. At group level, practice by observation led to sequence-specific gains in the performance of the observed movement sequence already after a 12-h interval, but the process was even more advanced, and occurred in more trainees, after an interval of 24 h. The current results, therefore, strengthen the notion that ‘how to’ knowledge gained in training by observation may not necessarily correspond to ‘how to’ knowledge acquired when the same task is practiced physically^18^. Repeated observation may initiate skill consolidation processes that are more alike to the processes triggered in perceptual learning, than the processes underlying the consolidation of ‘how to’ knowledge acquired in actual, physical practice.

## Materials and methods

### Participants

A total of 80 young, healthy, adults (19–30 years old) participated in the experiment. After given a detailed explanation of the aim and nature of the experiment and providing an informed consent, participants were asked to respond to three questionnaires: a personal health and life habits questionnaire; the Horne-Östberg Morningness-Eveningness Questionnaire (MEQ) and the Edinburg handedness inventory^52^. Only right-handed participants were included in this study. Potential participants with known learning disabilities or medical conditions that can potentially impair fine motor performance (chronic medication, neurological diseases, musculoskeletal diseases), musicians with more than 2 years of intensive practice of a musical instrument and professional typists, were excluded. Participants with extreme chronotype (scores > 70—extreme morning-types, or < 30—extreme evening-types, on the MEQ) were excluded as well. The study was approved by the Faculty of Education’s Human Experimentation Ethics Committee at the University of Haifa (054/16). All methods were performed in accordance with the relevant guidelines and regulations. All participants gave their written informed consent prior their participation in the study.

### Task

The motor task used in the current study was the finger-to-thumb opposition sequence (FOS) learning task^18,20,21,53^. To execute the task, participants were instructed to oppose the fingers of the left (non dominant) hand to the thumb in a given five movement sequence as fast and accurately as possible. Two sequences, (sequence A and B, see Fig. 1A) of equal length and complexity, each the reverse of the other, were used, with each participant randomly assigned one of the sequences for training. The two sequences consisted of identical component movements, hence were matched for the number of movements per digit, and differed only in the order of their component movements.

### Design and procedure

All participants observed one sequence (O-FOS; sequence A or B randomly assigned) while the second, mirror-reversed sequence was used as a novel, untrained sequence (U-FOS; sequence B or A) and introduced only before it’s performance was tested. Participants observed their assigned FOS performed by a left hand and were tested on performing the FOS with their left hand [in line with the^20,21^ protocol]. At the beginning of the training session the FOS was explicitly explained and demonstrated. The training constituted the observation of short video clips of a hand (left) repeatedly performing the task in a manner affording the observer a viewpoint of looking at his/her own hand. The video clips showed the hand of a skilled performer (female, no errors committed) executing the FOS (sequence A or B in separate clips) 16 times in a 30 s long block, i.e., the FOS tapped at a comfortable pace enabling the following of each individual component movement. The training session included 10 blocks separated by 30 s breaks (to simulate the time-course of the test) for a total of 160 repetitions (~ 15 min). During training and testing the participants sat comfortably with their left hand extended on a table top. The participants were instructed not to move their fingers during the training session, keep their hand still and focus on the video; the session was supervised by the instructor-examiner. Participants were explicitly instructed not to tap the sequence during the post-training interval.

Participants were randomly assigned into four groups (Fig. 1C). The participants assigned to groups Obs_T_Imm and Obs_T_24h observed the FOS training video in the morning (between 7 and 9 am) and were tested immediately after the termination of the observation session or on the following morning (the day after the observation session; 24 h interval) respectively. These two groups were used to acertain that delayed gains (rather than immediate post-training gains) were expressed as the result of the FOS observation session (a replication of^18^). Two additional groups, Obs_T_day and Obs_T_night, had identical training by observing the video clips but were tested after a 12 h interval. The participants in the Obs_T_day group trained in the morning (7–9 am) and were tested on the evening of the day of training. Participants in the Obs_T_night group trained in the evening (7–9 pm) and were tested on the morning of the following day. Participants in the Obs_T_Day and the Obs_T_24h groups were explicitly instructed not to nap on the day of training. No technological method (EMG) was used to test for muscle contractions during the observation condition; previous studies found no above-baseline muscle activity in mental practice or observation learning of finger movement sequences^25,53^.

The FOS performance test was comprised of four successive test blocks. Each test block was 30 s long (a 30 s break followed each test block) with participants instructed to perform the movement sequence (O-FOS, U-FOS) repeatedly "as fast and as accurately as possible" starting with an auditory "start" cue and ending with a corresponding "stop" cue. Participants’ performing hand was video-recorded from an angle affording good separation of the four finger movements^21^. For each test-block, two measures of performance were determined from the recordings: (1) the number of sequences performed correctly during the 30 s test-block—as a measure of speed; (2) the number of sequencing errors—as a measure of accuracy. Participants were first tested on the observed sequence (O-FOS) and immediately after, in the exact same manner, on the new, unobserved sequence (U-FOS).

The beginning of a sequence was identified by the small finger to thumb component movement of the sequence (Fig. 1A); all incorrect opposition movements within a single trial of a sequence were counted as a single error (i.e., the measure reflected the number of incorrect sequences). Participants were specifically instructed that each correct sequence has 5 component movements and that missing any and specifically the final or initial tap (4,4, respectively) is counted as an error. Participants were instructed that occasional errors should not be corrected and to continue with the task without pause as smoothly as possible. Visual feedback or information about the individual's performance was not afforded; however, participants were encouraged to "now, give it your best" before each block.

Note that in order to test the effect of training by observation per se (without the experience gained in the actual performance of the sequence during a performance test), we adopted the method that was used in a recent study^18^, wherein the performance of the trained (observed) FOS, after the observation session, was compared with that of a previously unobserved (specifically, the mirror-reversed) FOS. The logic behind our approach is that any testing of the performance of a movement sequence is in essence a learning experience; necessitating physical enactment and thus physical practice. Indeed, tests may constitute very effective means for improving subsequent recall and performance, i.e., ‘test enhanced learning’ (e.g.,^55^). Thus, a baseline performance measurement would be a physical practice opportunity and the ensuing pattern of results will not be ascribable to learning by observation per se; the performance gains would refer to a ‘mixed’ training (physical and observation) regime.

Previous studies (e.g.,^21,53,56^) have shown that the two mirror-reversed sequences are of equal difficulty (i.e., equal baseline performance) but the delayed performance gains accrued in actual physical training on one of the sequences are sequence specific (i.e., no delayed gains for the other sequence). The same was found to be the case for learning by observation^18^. Thus, rather than addressing ‘Mixed’ training of a FOS (that was shown to result in larger gains in performance compared to training by observation (only) (e.g.,^18^)) the participants in the current study, in all conditions, underwent observation training and were tested at different time-points after the observation session on the observed (trained) FOS and on an untrained, new, mirror-reversed FOS, that was introduced only before its performance was tested. Given that delayed gains in the FOS task are sequence specific, only the O-FOS was expected to gain after the immediate post-training test. In a way the baseline FOS performance for each person is represented by the performance for the untrained sequence; a difference in performance between the O-FOS and U-FOS afforded a measure of the O-FOS specific delayed gains.

### Statistical analyses

The analyses were designed as within-subject comparisons; comparing the performance of the two FOSs (O-FOS, U-FOS) at each time point (Test). All analyses were performed on raw data with the two measurements of performance: speed—the number of correctly performed sequences within a 30 s. long test-block and accuracy—the number of errors (incorrect sequences) performed within a 30 s long test-block. The data of all participants was tested for normalcy of distribution using a Kolmogorov–Smirnov test. Both the data for the O-FOS performance speed (number of correct sequences) (*p* = 0.2) and the data for the U-FOS (*p* = 0.08) were normally distributed in our sample. Because of the very few errors committed in all tests (a floor effect) the accuracy data did not show a normal distribution. Paired-sample t-tests comparing performance in the O-FOS to the U-FOS were run for each measure of performance (speed, accuracy) in each group, separately. Repeated-measures ANOVAs were run for performance speed, with FOS (O-FOS, U-FOS) as a within-subject factor and training groups/conditions as the between subject factor. The non-parametric chi-square test was used to compare proportions of improvers (trainees showing O-FOS > U-FOS) to non-improvers (O-FOS ≤ U-FOS). The Statistical Package for the Social Sciences (SPSS Statistics for Windows, Version 19.0; IBM Corp., Armonk, NY) was used.

## Data Availability

The dataset of the current study is available from the corresponding author on reasonable request.
